# Acceptability and usability of a patient portal for men with prostate cancer in follow-up care

**DOI:** 10.3389/fdgth.2022.1045445

**Published:** 2022-11-14

**Authors:** David O’Connor, Jane Frankland, Jo Watts, Hazel Brodie, Kevin Hamer, Claire Foster, Alison Richardson

**Affiliations:** ^1^ Informatics Department, University Hospital Southampton NHS Foundation Trust, Southampton, United Kingdom; ^2^ Centre for Psychosocial Research in Cancer, School of Health Sciences, University of Southampton, Southampton, United Kingdom; ^3^ Southampton Academy of Research, University Hospital Southampton NHS Foundation Trust, Southampton, United Kingdom

**Keywords:** patient portal, prostate cancer, remote monitoring, satisfaction, acceptability

## Abstract

**Background:**

A stratified approach to cancer follow-up care, including remote monitoring and supported self-management for suitable patients, is increasingly recommended. Patient portals can facilitate such an approach, allowing access to results and information. There is limited understanding of the use of portals within this context.

**Aim:**

This paper reports the acceptability and usability of a patient portal developed to facilitate a remote monitoring and supported self-management follow-up care programme for men with prostate cancer.

**Methods:**

A mixed methods evaluation, including analysis of service utilisation data, a survey of users' experiences and satisfaction, and telephone interviews of non-users' views and experiences.

**Results:**

Sixty percent of eligible patients registered to use the portal. Of these, 37% logged in at least once over a 6-month period and 52% over 12 months. Satisfaction among these users was reasonably high. Use of the portal in general was rated as very easy or easy by over 70% of respondents, and the majority felt the portal had helped them manage their condition in various ways. However, a large minority (40%) did not use the portal, with reasons for non-use including lack of access to computing facilities and lack of computer skills. Those who were older were less likely to register to use the portal.

**Conclusions:**

A large proportion of participants found the patient portal acceptable and easy to use. Reasons for non-use should be addressed in order to maximise system efficiencies and minimise inequalities in care, and the needs of specific groups should be taken into account.

## Introduction

The use of digital solutions for patient care management is undergoing rapid expansion internationally. Developments include electronic medical/health records, telemonitoring systems, online patient portals, web-based interventions and mobile apps. Such solutions are key to addressing increasing demand for health care (1) and have the potential to empower patients to take a greater role in the management of their condition (2, 3).

Patient portals are secure online websites (4) commonly tethered to a health care service's electronic medical record (EMR) and allow 24-hour patient access to personal health data, as well as functions such as secure messaging and patient information (5). In the United States, widespread use of internet accessible EMRs has been encouraged through the “Meaningful Use” regulation (6). In the United Kingdom (UK) summary information from primary care records has been available online to patients since 2016 (7), with full prospective record access planned for November 2022 (8), and stand-alone patient portals have been developed locally for patients with a variety of long-term conditions (9).

With improving survival rates, cancer is now considered a long-term condition. Post-treatment follow-up care to monitor for disease recurrence and to address treatment related side-effects continues for a number of years, but services are increasingly challenged to manage this activity for a rapidly growing population of patients. In the UK, a stratified approach to follow-up care is recommended, replacing routine face-to-face follow-up care appointments with remote monitoring and supported self-management for suitable patients (10). Patient portals can facilitate such an approach.

There has, to date, been limited evaluation of the use of patient portals in cancer populations (11, 12). A small number of studies have shown their introduction to be feasible and generally acceptable (13–16). Studies report uptake to be around 75–80% (14, 15), and users express high levels of satisfaction (17, 18). Where medical test results are made available to patients through a portal, this is the most used action (13, 14, 16, 17) and anxiety does not increase among patients when viewing results in this way (15, 16). There is appreciation of improved communication processes with the healthcare team (11). There is some evidence of improvement in patient reported outcomes, such as reductions in global distress (14), anxiety (15), in emotional and social functioning and mental health (16), and in physical activity (16). There is also some evidence of differential use, with greater use among people who are younger, white, with higher socio-economic status (11, 13, 19–21), and among men (13).

This paper reports the acceptability and usability of a patient portal developed to facilitate a remote monitoring and supported self-management follow-up care programme for men with prostate cancer.

## Materials and methods

### Development of the patient portal

As part of a global initiative to improve outcomes for men treated for prostate cancer, the Movember Foundation funded a service improvement initiative, the TrueNTH Supported Self-Management and Follow Up Programme, in five NHS Trusts in England from 2014 to 2017. The aim of the Programme was to redesign the post-treatment follow up care pathway, moving from a traditional model of clinic/office based follow up appointments to one of remote monitoring and supported self-management for men at low risk of recurrence and able to manage their own health needs. Evaluation of the impact of the Programme as a whole on patient reported outcomes and costs is reported elsewhere (22). The Programme has become normal practice at all five of the initial project sites.

**Figure 1:**
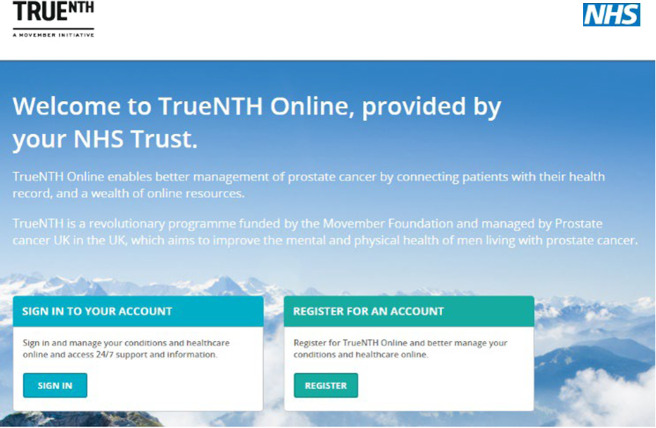
Screenshot of patient portal home page*. *Figure provided by Informatics Department, University Hospital Southampton NHS Foundation Trust.

The Programme is enabled through an integrated online system, which encompasses the patient portal alongside a healthcare professional accessed clinical monitoring system. The online system was designed by a working group involving clinicians, patients, academics, IT specialists and third sector representatives. The system uses a platform developed by Get Real Health™. It was piloted with two NHS organisations in 2014 and, following minor modifications, was rolled out to another three NHS organisations. Version 1 of the System was launched on 02/07/2014. Patients experienced difficulties with a complex registration process, so a second version with a modified process was launched on 20/10/2014. The system is managed by University Hospital Southampton NHS Foundation Trust through the Microsoft Azure cloud-based platform. A screen shot of the home page is shown in Figure 1.

Men are assessed for suitability for the Programme at their end of treatment appointment. If they meet the Programme criteria (which include assessment against defined clinical criteria, as well as symptom resolution and patient preference) they are introduced to the Programme by a Cancer Support Worker who, under supervision of a Clinical Nurse Specialist, manages the Programme on a day-to-day basis. Henceforth, men do not have scheduled clinic/office-based appointments, with their care being managed remotely. All men entering the Programme are registered on the system, since their care is managed through the clinical monitoring aspect of the system. This allows the clinical team to track patients’ follow-up care and test results and to run on-line “virtual clinics”, whereby they view details of all patients due for review, record their decisions about follow up care and communicate these to the patient. Blood tests for the marker of prostate cancer recurrence (Prostate Specific Antigen or PSA) are taken by the man's General Practice or hospital phlebotomy service and results transferred directly from the pathology laboratory to the online system. PSA values are monitored by the Support Worker and Clinical Nurse Specialist through the clinical monitoring system and men are only recalled to clinic if their results prompt further investigation. Men choose whether or not to register with the patient portal. They attend a four-hour workshop to introduce them to the Programme and during this session can view the patient portal and ask questions. All men also receive letters detailing their test result and have easy access to the clinical team by telephone, ensuring that men who choose not to register on the portal still have access to information and support.

The patient portal has four broad functions:

i) The PSA results and tracker functionality presents results to patients as soon as available, often within a few hours of the blood test being taken. Previous PSA results are held on the system and presented on a line graph to allow comparison over time.
ii) An e-mail style messaging service allows for secure messaging between patient and clinician. Messages are internal to the system and can only be viewed from within it. Notifications are sent to patient and clinicians' external e-mail accounts prompting them to log in and view the message. The messaging service allows patients to contact their clinical team with any questions or concerns without having to telephone or visit the hospital, while allowing the clinicians to respond to messages when appropriate and convenient.
iii) A Health MOT (holistic needs assessment or HNA) comprises a questionnaire completed by the patient within the portal. HNAs ask about emotional, practical, financial and clinical concerns in order to facilitate personalised care (23). Men are asked to complete regular HNAs at the same intervals as their PSA tests, indicating if they would like input from their clinical team. Men can choose to complete an additional HNA at any other time.
iv) A patient information function provides an online space for validated information about prostate cancer, in the form of documents, links to other websites and film clips. This includes information on managing side effects and consequences of cancer, healthy lifestyles and support for computer/IT skills.

### Study design

A mixed methods design was used to assess the experiences of both users and non-users of the portal. This included analysis of utilisation data, an online survey of the experience and satisfaction of those who chose to have access to the patient portal, and structured telephone interviews of non-users' views and experiences. The evaluation took place between July and September 2017, three years after introduction of the portal into the prostate cancer follow up care pathway. All patients at the five NHS Trusts registered on the Programme on 31 August 2017 were included.

### Data collection

#### Portal utilisation data

Portal utilisation data were extracted using bespoke data extracts. The data items for each patient included date registered on the Programme, NHS Trust, whether they were registered to use the portal, and how many times they had logged into the portal during the 6 months and 12 months prior to 31 August 2017. Data relating to website traffic and use of the key functions of the portal (patient information, clinician messaging, PSA results, and Health MOT) were collected anonymously using Google Analytics.

#### Portal user survey

An online survey to assess users' experiences of the portal was sent to all registered patients who had logged onto the portal more than once over a 28-month period from 1 January 2015 to 30 April 2017. A link to the survey was e-mailed to these users on 17 August 2017 (allowing time for those who registered during the latter months to form an opinion of the portal). The survey was open for a period of 29 days. Questions were asked about views and use of the portal; support for use; and impact on care management. The survey included various question types including list, category, ranking, quantity and grid, along with free text boxes to allow respondents to expand on their responses (see Supplementary Material 1). A net promoter score (NPS) (24) was included. The NPS is determined by asking respondents to score: “how likely would you be to recommend the IT Service to other patients?” on a scale of 0 = “Not at all likely” to 10 = “Extremely likely”. Respondents scoring 0–6 are classed as detractors (unhappy), 7–8 as passives (satisfied), and 9–10 as promoters (enthusiasts). The NPS is calculated by subtracting the proportion of detractors from the proportion of promoters. In general, a positive NPS is considered good, anything over 50 excellent and 70–80 + world class. The questionnaire was piloted with two patients and refined based on their feedback. The survey was anonymous and took between 5 and 10 min to complete.

#### Structured telephone interviews with non-users of the portal

Structured telephone interviews were conducted with two groups of patients at the host Trust who were registered on the Programme on 31/12/2016 but not actively using the portal: those who had chosen not to register to use the patient portal; and those who had registered to use the portal but had never logged in or had logged in only once. Questions to these groups included reasons for non-use, awareness of support and general computer usage. Patients considered too unwell by the clinical team to be approached were excluded. Trained interviewers conducted the interviews, which took approximately five minutes each and followed a structure template. The interviews were conducted between 24 August 2017 and 19 September 2017.

#### Ethical issues

The study met the UK NHS Health Research Authority and University Hospital Southampton NHS Foundation Trust's (UHSFT) Service Evaluation Guideline criteria for classification as service evaluation. University Hospital Southampton NHS Foundation Trust's (UHSFT) Service Evaluation Guideline does not require approval from a research ethics committee. Survey participants were informed by email and at the start of the survey about the purpose of the study and that responses would be anonymous. Consent to participate was assumed by survey completion. Interview participants were given information about the purpose and voluntary nature of the interviews by text, with an option to opt out. At the start of an interview, the interviewers explained the same information, and asked for verbal consent to take part. Approval was sought from each NHS Trust via the lead clinician, using processes required by each site where the evaluation took place. Data were managed in line with information governance and data collection policies.

#### Data analysis

Utilisation data and web page views were summarised descriptively, using Microsoft Excel. *χ*^2^ tests were employed to explore differences in patient registration by age and by NHS Trust. To explore the nature of any relationship between patient age and portal registration/use, data for percentage registration and use over twelve months were plotted against midpoint age range on a scatter graph. A straight line of best fit along with the associated R score (Pearson's correlation coefficient) and *R*^2^ score (co-efficient of determination) were calculated. All analyses were conducted using Microsoft Excel.

The results of the survey were summarised descriptively using Microsoft Excel. Free text responses were coded by a researcher experienced in qualitative coding, using the NVivo software (25). The coding followed the “Eclectic” approach set out by Saldana (26). This approach is a hybrid method that can be used for a wide variety of qualitative data forms. A deductive/inductive approach was used, starting with *a priori* codes taken from the survey questions and then adding codes that emerged inductively during the process. The free text comments were coded to topics (e.g., look and feel, logging on, PSA results, website navigation), evaluations (e.g., positive, negative, satisfactory, fast, slow), emotions (e.g., reassurance and confidence, reducing anxiety and stress) and suggestions of what to stop, start and continue, as appropriate. Matrix coding queries were used to analyse and cross reference the topics with the evaluations, emotions and suggestions of what to stop, start and continue. Themes were developed from the matrix coding analysis.

Results of the structured telephone interviews with non-users were summarised descriptively in Microsoft Excel.

## Results

### Portal utilisation

Sixty percent (1,556/2,599) of those who were eligible chose to register to use the portal (see Table 1 and Supplementary Material 2 Figure S1). The analysis of user log-in data (Table 1) shows that 37% (575/1556) of those registered had logged in at least once over a 6-month period and 52% (810/1556) over a 12-month period. Most patients tended to access the portal when they were expecting a test result (41%), and only 12.5% accessed it once a month or more (Table 2).

**Table 1:** Portal registration and use.

Site	Total men registered on Programme *N* (%)	Registered to use the portal			Not registered to use the portal *N* (% of users at site)
		Total *N* (% of total at site)	Logged in during 6 months to 31/08/17 *N* (% of users at site)	Logged in during 12 months to 31/08/17 *N* (% of total at site)	
**A**	481 (18.5)	332 (69.0)	133 (40.1)	194 (58.4)	149 (31.0)
**B**	905 (34.8)	421 (46.5)	182 (43.2)	225 (53.4)	484 (53.5)
**C**	257 (9.9)	187 (72.8)	85 (45.5)	117 (62.6)	70 (27.2)
**D**	691 (26.6)	505 (73.1)	126 (25.0)	199 (39.4)	186 (26.9)
**E**	265 (10.2)	111 (41.9)	49 (44.1)	75 (67.6)	154 (58.1)
**Total (all sites)**	**2,599** **(100)**	**1,556** **(59.9)**	**575** **(37.0)**	**810** **(52.1)**	**1,043** **(40.1)**

**Table 2:** Frequency of Portal use.

	Responses *N* (%)
Less than once every three months	88 (17.0)
Once every three months	135 (26.1)
Once a month	41 (7.9)
Two to three times a month	15 (2.9)
Once a week or more	9 (1.7)
Only when I am expecting a test result	213 (41.1)
Missing	17 (3.3)
**Total**	**518** **(100.0)**

**Figure 2:**
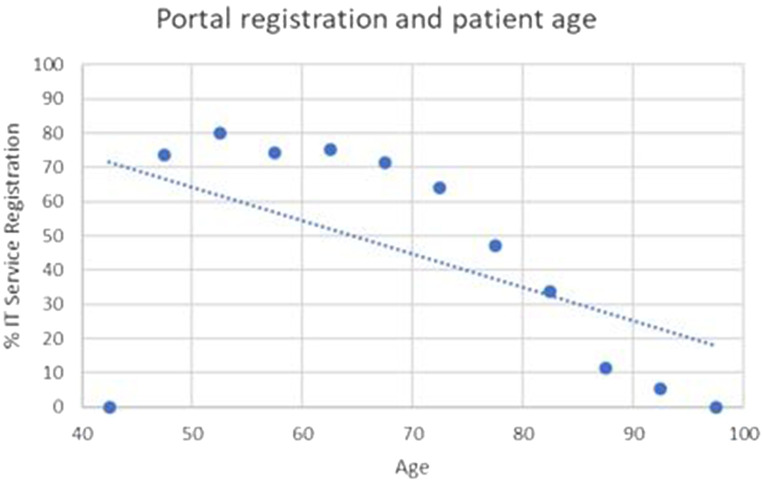
Portal registration by age.

Figure 2 shows the percentage of portal registration rates by age. There was a strong correlation between patient age and portal registration [*χ*^2^ (11, *N* = 2,599) = 122.73, *p* < 0.001], with older patients less likely to be registered [*r* (2,598) = −0.94, *p* < 0.001, *R*^2^ = 0.88, excluding single outlier point at 42.5, 0]. There was also a statistically significant variation in patient registration by NHS Trust [*χ*^2^ (4, *N* = 1,556) = 75.15, *p* < 0.001] (Table 1). This influence remained when controlling for inter-trust patient age (see Supplementary Material 2 Tables S1–S3).

**Figure 3:**
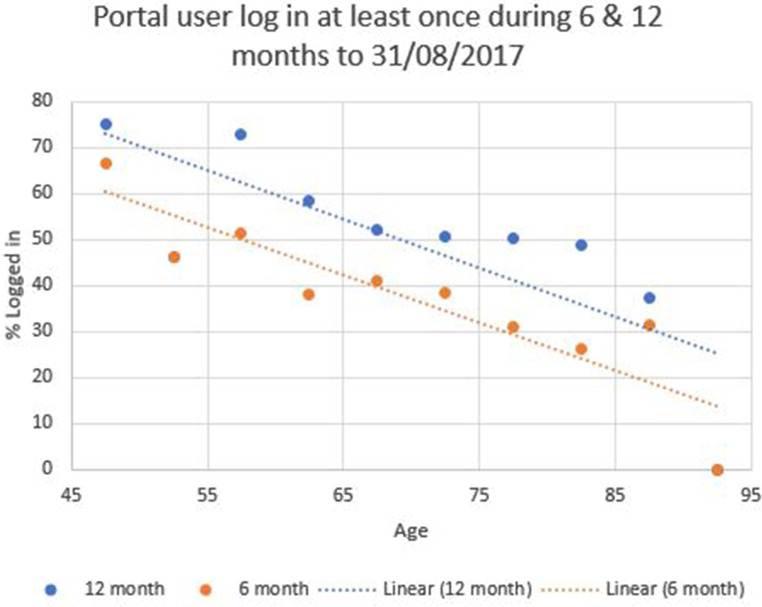
User log in over prior 6- and 12-month period.

There was a weaker, but still significant, correlation (Figure 3) between age of patient and log in over the prior 6 months (*χ*^2^ (9, *N* = 1,556) = 17.46, *p* = 0.04. and *r* (1,556) = −0.90, *p* < 0.01, *R*^2^ 0.81), with older patients less likely to have logged in. The correlation between age of patient and log in over the prior 12 months (Figure 2) was not statistically significant (*χ*^2^ (9, *N* = 1,556) = 12.22, *p* = 0.2. and *r* (1,556) = −0.78, *p* < 0.01, *R*^2^ 0.61).

Table 3 shows web page views for the key functions of the portal over a six-month period. Men most commonly used the portal to view PSA test results pages (46.7% of views), while the patient information and Health MOT pages were viewed the least (14.5% and 14.3% of views respectively).

**Table 3:** Portal web page views over a 6 month period.

Portal Main Patient Functions Page Views (1 March to 31 August 2017)
**Function**	*N*	**%**
PSA Results Tracker	4,275	46.7
Messaging	2,245	24.5
Patient Information	1,329	14.5
Health MOT	1,305	14.3
**Total**	**9,154**	**100**

### Portal user acceptability and usability

A response rate of 49% (*N* = 518) was achieved for the user survey (Supplementary material 2 Figure S1). Users were fairly satisfied with the portal. The Net Promoter Score (24) was 49 (*n* = 447), with 15% scored as detractors, 20% as passive and 64% as promoters (Supplementary material S2 Tables S4, S5). Four percent (*n* = 22) of respondents scored the portal as 0 on the NPS.

The portal's key functions (finding patient information, messaging the clinical team, checking PSA results and completing a Health MOT) were each rated as very easy or easy to use by over 70% of users (Table 4). Ratings of ease of conducting practicalities such as registering, logging in and changing password were lower.

**Table 4:** Portal ease of use.

	Very difficult *N* (%)	Difficult *N* (%)	Neither difficult or easy *N* (%)	Easy *N* (%)	Very easy *N* (%)
Use of the portal in general	19 (3.9)	23 (4.8)	94 (19.5)	166 (34.4)	181 (37.5)
Register to use the IT Service	20 (4.3)	48 (10.4)	86 (18.6)	168 (36.3)	141 (30.5)
Log in	28 (6.1)	36 (7.8)	76 (16.5)	145 (31.5)	176 (38.2)
Change your password	18 (7.1)	19 (7.5)	60 (23.8)	75 (29.8)	80 (31.7)
Find patient information	17 (3.7)	32 (6.9)	85 (18.4)	162 (35.0)	167 (36.1)
Message your clinical team	18 (4.5)	16 (4.0)	60 (15.1)	140 (35.3)	163 (41.1)
Check your PSA results	25 (5.2)	11 (2.3)	35 (7.3)	162 (33.8)	247 (51.5)
Complete a Health MOT	21 (5.6)	20 (5.3)	58 (15.5)	135 (36.0)	141 (37.6)

**Figure 4:**
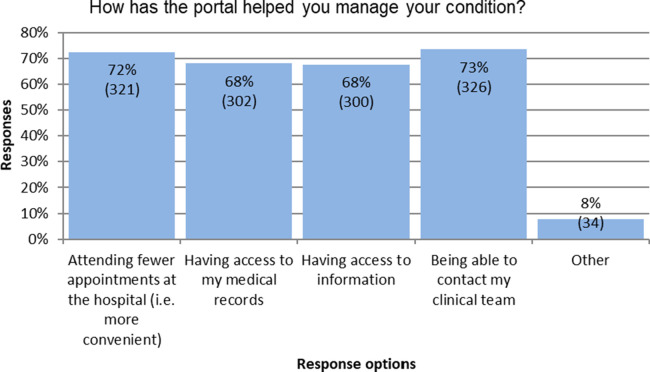
Ways the portal helped users their condition.

The portal functions were rated by 78%–91% of respondents as helpful or very helpful in managing their condition (Table 5). The most helpful functionality was access to PSA results (considered very helpful or helpful by 91% of respondents), followed by messaging the clinical team (87%). Although the survey responses were generally very positive, between four and nine percent of respondents reported that the various functions of the portal did not help them to manage their condition. The portal had helped users (Figure 4) by allowing them to attend fewer hospital appointments (72%), and by facilitating access to their medical records (68%), to information about their condition (68%), and contact with their medical team (73%).

**Table 5:** Aspects of portal that helped users manage their condition.

	Not helpful *N* (%)	Somewhat helpful *N* (%)	Helpful *N* (%)	Very helpful *N* (%)
Patient information	30 (7.7)	57 (14.6)	163 (41.7)	141 (36.1)
Messages to or from your clinical team	25 (7.2)	21 (6.1)	129 (37.3)	171 (49.4)
PSA results	19 (4.3)	19 (4.3)	116 (26.2)	288 (65.2)
Health MOT	31 (9.1)	35 (10.3)	120 (35.2)	155 (45.5)

Open ended responses to the survey gave additional detail about the value of the portal for users. A total of 963 responses were received from 518 participants. Respondents reported the PSA result and tracking function to be quick, convenient, saving on time travelling to the hospital, providing peace of mind and reducing stress and anxiety, and the clinician messaging function to be quick, easy and providing peace of mind.

“*Very quick information on PSA results gives me peace of mind. Clinical team can get in touch easily if required, and I can contact them quickly if needed. A very useful system much appreciated by me. Thank you very much.”*

*“System excellent when contact with team is required. Answers to queries answered very promptly. This is the real strength of the system and provides much peace of mind”*

Open-ended responses also shed light on problems men were having with the portal, including difficulties with the log-in process, problems with passwords and access, difficulties of compatibility with some operating systems and lack of support for use. A number of men indicated that their infrequent use of the portal compounded issues of ease of use and navigation.

*“because I only use it every 6 months I can never remember how it works so it takes ages to access account”*

A small number of respondents mentioned the impersonal nature of the portal, a preference for face-to-face clinical contact, and a lack of appropriateness of an IT solution for patients with low IT literacy. Conversely, a few respondents mentioned a desire for the portal to expand to include comprehensive medical reports and links/incorporation of other clinical issues.

### Understanding non-use of the portal

Telephone interviews were conducted with 38 men who chose not to register to use the portal, and with 10 men who had registered but then did not used the portal (Supplementary Material 2 Figure S1).

The reasons given by men who had registered but not used the portal (Supplementary Material 2 Table S6) included lack of access to a computer or the internet (*n* = 4) and lack of computer skills (*n* = 2). Suggestions of what might have encouraged their use included availability of support (*n* = 5), easier log in processes (*n* = 4), and the loan of IT equipment (*n* = 2). Five participants said that there was nothing that could be done to help as they did not want to use the portal.

Among those who had chosen not to register with the portal at all (Supplementary Material 2 Table S7), reasons included lack of access to a computer (*n* = 10), not wanting to use computers for healthcare (*n* = 8), not liking to use computers (*n* = 5) and lack of access to the internet (*n* = 2). The majority of this group stated that nothing could be done to encourage use of the portal (*n* = 28), although five suggested more support might help.

## Discussion

IT and digital solutions have a major part to play in the remote medical management of patients and in the delivery of personalised care (27, 28). The development and use of such solutions has been accelerated by the COVID-19 pandemic (29). Patient portals have the potential to support self-management, with access to personal health data and the ability to interact with care providers, empowering patients to take control of their health and care (27, 30). Combining patient portals with clinician facing monitoring systems to enable remote monitoring and supported self-management is a way of addressing overburdened health services, containing costs, and improving health outcomes through enhanced patient involvement (10).

This study considered the acceptability and usability of a patient portal which is part of a cancer remote monitoring and supported self-management follow-up care pathway. The portal was a feasible option and was acceptable to the majority of men registered on the Programme. Most of those who opted to use it found the functions easy to use and valuable in management of their prostate cancer follow-up care, through access to test results, easy contact with their clinical team and access to information. There was a number, however, who found practicalities such as registering, logging in and changing passwords to be difficult, which highlights a danger that people can be lost to the system early on without support for these issues. These findings add to a growing body of evidence regarding the feasibility, acceptability and usefulness of patient portals among people living with and beyond cancer (13, 15, 16).

There was a low frequency of use, with users often only logging in to view test results, and low use of other functions such as information pages. Low use in this study is likely to be partly related to these patients being in their post treatment follow up phase and thus moving on from cancer. However, this type of usage has been found across other studies (13, 17). For instance, in a study with a mixed group of cancer patients, most portal use was for viewing test results, then messaging the clinical team, with low use of information functions (13). Low use of functions such as information suggests that development of such features should be personalised and designed with input from users in order to ensure they meet needs and preferences.

There was a large minority of men offered access who chose not to use the portal. The study gave some insights into reasons for non-use, with lack of IT experience being commonly cited. Other studies have similarly reported low uptake (14, 31), with reasons for low use including concerns about security, lack of guidance on use, and inability to understand information (32, 33). In the present study, older men were less likely to make use of the portal: they were less likely to register or to use the portal over a 6-month period. It is known that internet access is increasingly less among older populations, in particular among the oldest (34), and other studies have shown similar trends in portal use by age (13, 35). A study of age differences in eHealth literacy and use of technology among cancer patients (20) found older people to have lower eHealth literacy, and to be less confident in use of internet health information, and less likely to own a smart phone, to have an email address or to use a patient portal. In addition, younger people are likely to prefer a more active role in their health care (36) and, as such, might be more likely to use the portal for functions other than viewing their test result. There also was variation in uptake by cancer centre. It may be that differing levels of encouragement and support from the clinical teams could have contributed to these differences. Perception of the value of an intervention is important for implementation, and studies have found low uptake related to lack of perceived benefit (31, 33, 37, 38). Perceived value in this study may have been reduced because men on the Programme were also able to access test results in other ways, such as by letter.

There are concerns that digital solutions may contribute to health inequalities (3). In addition to older age, other social factors such as ethnicity (21, 35) and social deprivation (21) have been associated with lower use. Barriers to use and ways to maximise accessibility, such as through mobile applications (39) need to be considered. While providers can promote the value of access to results through the portal, other routes need to be maintained in order to accommodate those without computer/internet access.

Conceived as a pragmatic evaluation, the focus of the work reported here was on satisfaction, reach and acceptability of the portal in order to inform its development. While this evaluation did not relate patient outcomes to portal use, an evaluation of the impact on patient reported outcomes of the whole follow-up care pathway, of which the portal is a part, has shown this type of follow-up care to be broadly comparable to traditional clinic based care in terms of patient reported outcomes and costs (22). Since the evaluation was conducted, there has been increasing interest in the use of patient portals across long term conditions, but there are still only a small number of published studies which address these issues of feasibility and acceptability for people living with and beyond cancer, and fewer still that address non-use of a portal. This paper is thus an important contribution to this literature in terms of understanding acceptability, reach and user satisfaction in this context. The portal has undergone some minor refinement, mainly to the user interface (partly in response to the findings of this study) but continues with the same key functions. The portal continues to be used by a growing number of men at the five initial project sites. The online management system and portal is also made available to other interested cancer centres around the UK, although many UK NHS Trusts still do not offer patient portals as part of cancer follow-up care.

The lessons learned from this study make an important contribution to our understanding of models of virtual cancer follow-up care, indicating acceptability and usability of a digital portal as part of this model. It also contributes to the wider literature on portal use for the management of long-term conditions generally. It has contributed to understanding reasons for non-use which, if addressed, could help to minimise potential inequalities in care. The study indicates a need for services to be flexible in their expectations of patient uptake and use of a portal and its varied functions. While many of the lessons learned are likely to be relevant to such services for other types of cancer patients, and for other long-term conditions, there is a need for clinician and user input into design for each specific group of patients.

## Data Availability

The datasets presented in this article are not readily available because **this was a service evaluation**. Requests to access the datasets should be directed to j.l.frankland@soton.ac.uk.
