# Supplementary material for: Acceptability and usability of a patient portal for men with prostate cancer in follow-up care
Source: Front Digit Health. 2022 Nov 14;4:1045445. doi: 10.3389/fdgth.2022.1045445 (PMC9703137; doi:10.3389/fdgth.2022.1045445)
Supplement: Supplementary file 1 [file Datasheet1.pdf]

IT Service questionnaire

Welcome to the IT Service Questionnaire

You have registered to use an IT Service as part of your prostate cancer care. We are undertaking a survey of patients who use the IT Service.

We are keen to understand the experiences of those who use it and how we might improve it.

Please can you help us by completing a short questionnaire about the IT Service?

This survey is anonymous. No one will be able to identify you or your answers, and no one will know whether you participated in the study.

The survey should take 5 to 10 minutes to complete.

About you

1. Which of the age ranges below best describes you?

We are committed to supporting people of all ages in using our services, therefore it is useful to know your age range.

- Under 30
- 30-34
- 35-39
- 40-44
- 45-49
- 50-54
- 55-59
- 60-64
- 65-69
- 70-74
- 75-79
- 80-84
- 85-89
- 90-94
- 95-99
- 100+
- Prefer not to answer

2. Which NHS Trust are you receiving your care from?

- [redacted]
- [redacted]
- [redacted]
- [redacted]
- [redacted]
- Other / Do not know

Using the IT Service

3. What type of computers / IT devices do you use to connect to the IT Service?

Click all that apply

- [ ] Desktop (including iMac, Mac Mini, Mac Pro etc.)
- [ ] Laptop (including MacBooks)
- [ ] Tablet
- [ ] iPad
- [ ] Smartphone
- [ ] Other (please specify)

4. How often do you use the IT Service?

- ○ Less than once every three months
- ○ Once every three months
- ○ Once a month
- ○ Two to three times a month
- ○ Once a week or more
- ○ Only when I am expecting a test result (every three months/ six months/ twelve months)

5. How long have you been using the IT Service?

- ○ Less than 12 months
- ○ 12 to 24 months
- ○ 25 to 36 months
- ○ More than 36 months

Ease of use

6. On a scale of 1 to 5, where 1 is very difficult and 5 is very easy, how is it to:

	1 very difficult	2 difficult	3 neither difficult or easy	4 easy	5 very easy	N/A
Use the IT Service in general?	◯	◯	◯	◯	◯	◯
Register to use the IT Service?	◯	◯	◯	◯	◯	◯
Log in?	◯	◯	◯	◯	◯	◯
Change your password?	◯	◯	◯	◯	◯	◯
Find patient information?	◯	◯	◯	◯	◯	◯
Message your clinical team?	◯	◯	◯	◯	◯	◯
Check your PSA results?	◯	◯	◯	◯	◯	◯
Complete a Health MOT? (The Health MOT is an assessment your clinical team may have asked you to complete)	◯	◯	◯	◯	◯	◯

7. Please provide details of any problems that you have had with these aspects of the IT Service.

Features

8. What section of the IT Service do you use the most?

Please rank this list of features in order of how often you use them

- [ ] Messaging your clinical team
- [ ] PSA results
- [ ] Health MOT
- [ ] Patient Information

9. Please list other features you would like to see added to the IT Service and how useful they would be

Look and feel

10. What do you think about how the IT Service looks?

- ◯ Well designed – doesn’t need any changes
- ◯ Acceptably designed – could do with a few minor changes
- ◯ Adequately designed – needs quite a few changes
- ◯ Poorly designed – needs major improvement

11. What would improve how it looks and works for you?

IT support (getting started and ongoing use)

12. You may have attended a Supported Self-Management workshop run by your clinical team as part of your care. Did the workshop help you to use the IT Service?

- ◯ Workshop did not cover using the IT Service
- ◯ Workshop covered using the IT Service, it was not helpful
- ◯ Workshop covered using the IT Service, it was somewhat helpful
- ◯ Workshop covered using the IT Service, it was very helpful
- ◯ Not applicable. I did not attend a Supported Self-Management workshop

IT support (getting started and ongoing use)

13. Did you require additional help to use the IT Service in the first three months of when you first started using it, and if so who helped you?

	No, I didn't need help	Yes, from my clinical team (eg. a support worker)	Yes, from a family member	Yes, from a friend	Yes, from a paid carer	Other	N/A
Use the IT Service in general?	○	○	○	○	○	○	○
Register to use the IT Service?	○	○	○	○	○	○	○
Log in?	○	○	○	○	○	○	○
Change your password?	○	○	○	○	○	○	○
Find patient information?	○	○	○	○	○	○	○
Message your clinical team?	○	○	○	○	○	○	○
Check your PSA results?	○	○	○	○	○	○	○
Complete a Health MOT?	○	○	○	○	○	○	○

14. Please provide details of any other sources of help

15. Do you currently require help to use the IT Service, and if so who helped you?

	No, I don't need help	Yes, from my clinical team (eg. a support worker)	Yes, from a family member	Yes, from a friend	Yes, from a paid carer	Other	N/A
Use the IT Service in general?	○	○	○	○	○	○	○
Register to use the IT Service?	○	○	○	○	○	○	○
Log in?	○	○	○	○	○	○	○
Change your password?	○	○	○	○	○	○	○
Find patient information?	○	○	○	○	○	○	○
Message your clinical team?	○	○	○	○	○	○	○
Check your PSA results?	○	○	○	○	○	○	○
Complete a Health MOT?	○	○	○	○	○	○	○

16. Please provide details of any other sources of help

How you use the IT Service

17. Do you print out, or would you like to be able to print any of the following?

	Yes, I print	No, but I would like to print and I **have** a printer	No, but I would like a print out but I **do not have** a printer	No, I do not want to print	N/A
Patient information?	○	○	○	○	○
Messages to or from your clinical team?	○	○	○	○	○
PSA results?	○	○	○	○	○
Health MOT?	○	○	○	○	○

18. Any comments on printing?

Problems

19. Do you think the IT Service is reliable?

- ◯ Extremely reliable – always works when I use it
- ◯ Mostly reliable
- ◯ Reliable
- ◯ Sometimes unreliable
- ◯ Often unreliable

IT Service questionnaire

Do you think the IT Service has helped you manage your condition?

20. Has the IT Service helped you manage your condition:

	Not helpful	Somewhat helpful	Helpful	Very helpful	N/A
Patient information?	○	○	○	○	○
Messages to or from your clinical team?	○	○	○	○	○
PSA results?	○	○	○	○	○
Health MOT?	○	○	○	○	○

21. How has the IT Service helped you manage your condition?

Click all that apply

- [ ] Attending fewer appointments at the hospital (i.e. more convenient)
- [ ] Having access to my medical records
- [ ] Having access to information
- [ ] Being able to contact my clinical team
- [ ] Other (please specify)

22. Do you have any further comments in relation to how the IT Service has helped you manage your condition?

IT Service questionnaire

Do you think the IT Service has helped you manage your condition?

23. How likely would you be to recommend the IT Service to other patients?

0 = Not at all likely, 10 = Extremely likely

0	1	2	3	4	5	6	7	8	9	10
○	○	○	○	○	○	○	○	○	○	○

24. Are there any other comments you would like to make about the IT Service?
