# Supplementary material for: Acceptability and usability of a patient portal for men with prostate cancer in follow-up care
Source: Front Digit Health. 2022 Nov 14;4:1045445. doi: 10.3389/fdgth.2022.1045445 (PMC9703137; doi:10.3389/fdgth.2022.1045445)
Supplement: Supplementary file 2 [file Datasheet2.pdf]

Supplementary Material 2

FIGURE 1: STUDY FLOW CHART

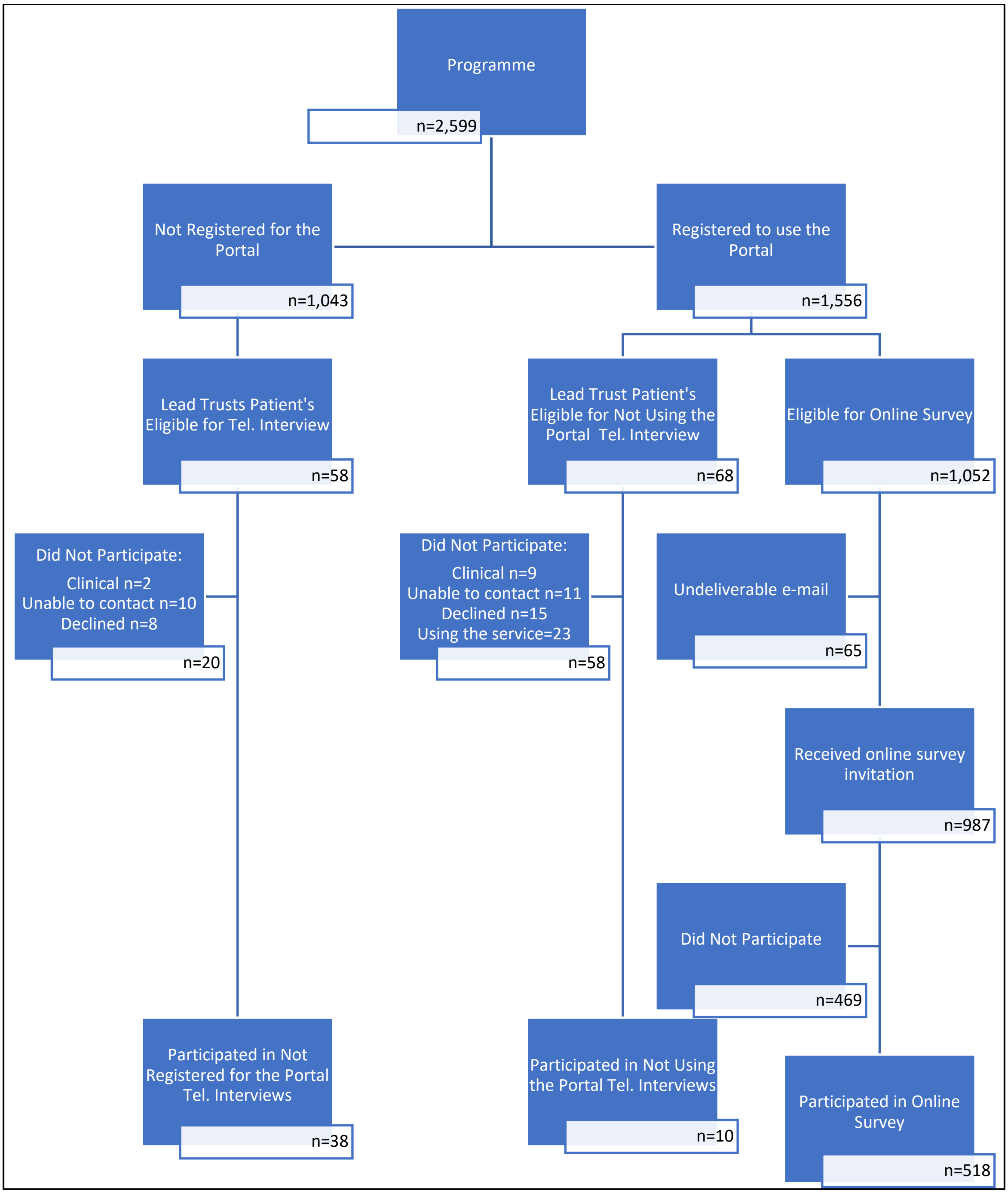

Table 1: Programme patient age and NHS Trust data

NHS Trust	Age in years								
	Mean	Median	Std Dev	Min	Max	Range	Q1	Q3	IQR
Site A	70	70	7	47	93	46	66	75	9
Site B	73	73	8	45	96	51	68	79	10
Site C	69	69	8	46	91	45	63	74	10
Site D	71	72	7	44	91	47	67	76	9
Site E	74	75	7	48	93	45	71	79	9
**All Patients**	**72**	**72**	**8**	**44**	**96**	**52**	**67**	**77**	**10**

Table 2: Portal adoption and NHS Trust data controlled for patient age

NHS Trust	Total on Programme (N)	Actual Portal Patients N (%)	Expected Portal Patients (controlled for age) N (%)
**Site A**	481	332 (69.0)	302 (62.7)
**Site B**	905	421 (46.5)	504 (55.7)
**Site C**	257	187 (72.8)	167 (65.0)
**Site D**	691	505 (73.1)	441 (63.8)
**Site E**	265	111 (41.9)	142 (53.6)
**All Patients**	**2599**	**1556 (59.9)**	**1556 (59.9)**

Table 3: Chi squared test for portal adoption and NHS Trusts data controlled for patient age

Chi Squ Test Stat	35.17
p	$4.29\text{x}10^{-07}$
95% critical value	9.49
99% critical value	13.28

Table 4: Portal Net Promoter Score (NPS) results

How likely would you be to recommend the IT Service to other patients?<br>0 = Not at all likely, 10 = Extremely likely												
**Score**	**0**	**1**	**2**	**3**	**4**	**5**	**6**	**7**	**8**	**9**	**10**	**Total**	**Skipped**
**N (%)**	22 (4.9)	4 (0.9)	1 (0.2)	5 (1.1)	4 (0.9)	20 (4.5)	13 (2.9)	19 (4.3)	72 (16.1)	53 (11.9)	234 (52.4)	447 (100.1)	71

TABLE 5: PORTAL NPS CALCULATION

NPS	
	**N (%)**
Detractors	69 (15.5)
Passive	91 (20.4)
Promoters	287 (64.3)
Missing	71 (13.7)
**Total**	**518 (100.0)**
**NPS**	**48.8**

Table 6: Reasons for non-use of portal – users who were registered but not using the portal

**Q1. "You were invited to use an IT service called My Medical Record to manage your prostate cancer. You registered but have not used the service. We would like to understand why?"**	**Total**
Too difficult	2
Would like more training	0
Can't remember my password	2
Don't have access to the internet	1
Don't have access to a computer (e.g. broken and not repaired, I don't have one anymore)	3
Don't have access to the internet anymore (no broadband access, no data allowance on mobile devices)	0
The IT Service is not in a language that I am confident with (e.g. lack of proficiency in reading/writing English)	0
It doesn't work well on my devices (e.g. not optimised for my tablet)	0
I don't trust the internet	1
Did not answer	0
**Q2. "What could we do to make it more likely that you would use the IT Service?"**	
Nothing – I don't want to use it?	5
Make the log in process easier?	4
Provide a face to face session on my own helping me to use it	3
A telephone call to talk me through how to use it	2
Provide a loan computer/ device with internet access	2
Other type of support (please ask them to give details)	0
Did not answer	7

TABLE 7: REASONS FOR NOT REGISTERING ON THE PORTAL

**Q1. "You were invited to use an IT solution to manage your prostate cancer. You have declined to use it and we would like to understand why?"**	
I don't have a computer	10
I don't have internet access	2
I don't like using my computer	5
I don't want to use computers for my healthcare	8
It would be too difficult for me	5
The IT Service is not in a language that I am confident with (e.g. lack of proficiency in reading/writing English)	0
Other	14
Did not answer	1
**Q1a. Only ask this question if they told you that they have not got a computer. "Please can you tell me the reason(s) you don't have a computer?"**	
Not interested in using a computer	5
Would rather spend my money on other things	0
Don't want to learn something new	1
Too old for computers	3
Other	1
Have computer (question not asked)	26
Did not answer	2
**"What would encourage you to use an IT service as part of your care?"**	
Nothing	28
Provide a computer for me to access it	0
Explain to me how you keep my personal data safe	0
More support to use it	5
Other	5
Did not answer	2
